# Dysregulated microRNAs in prostate cancer: *In silico* prediction and *in vitro *validation

**DOI:** 10.22038/IJBMS.2024.75164.16299

**Published:** 2024

**Authors:** Samaneh Rezaei, Mohammad Hasan Jafari Najaf Abadi, Mohammad Javad Bazyari, Amin Jalili, Reza Kazemi Oskuee, Seyed Hamid Aghaee-Bakhtiari

**Affiliations:** 1 Department of Medical Biotechnology and Nanotechnology, Faculty of Medicine, Mashhad University of Medical Sciences, Mashhad, Iran; 2 Bioinformatics Research Center, Mashhad University of Medical Science, Mashhad, Iran

**Keywords:** Computational biology, MicroRNA, Prostatic neoplasm, Therapeutic biomarker, Therapeutics

## Abstract

**Objective(s):**

MicroRNAs, which are micro-coordinators of gene expression, have been recently investigated as a potential treatment for cancer. The study used computational techniques to identify microRNAs that could target a set of genes simultaneously. Due to their multi-target-directed nature, microRNAs have the potential to impact multiple key pathways and their pathogenic cross-talk.

**Materials and Methods:**

We identified microRNAs that target a prostate cancer-associated gene set using integrated bioinformatics analyses and experimental validation. The candidate gene set included genes targeted by clinically approved prostate cancer medications. We used STRING, GO, and KEGG web tools to confirm gene-gene interactions and their clinical significance. Then, we employed integrated predicted and validated bioinformatics approaches to retrieve hsa-miR-124-3p, 16-5p, and 27a-3p as the top three relevant microRNAs. KEGG and DIANA-miRPath showed the related pathways for the candidate genes and microRNAs

**Results:**

The Real-time PCR results showed that miR-16-5p simultaneously down-regulated all genes significantly except for *PIK3CA/CB *in LNCaP; miR-27a-3p simultaneously down-regulated all genes significantly, excluding MET in LNCaP and PIK3CA in PC-3; and miR-124-3p could not down-regulate significantly *PIK3CB*, *MET*, and *FGFR4* in LNCaP and *FGFR4* in PC-3. Finally, we used a cell cycle assay to show significant G0/G1 arrest by transfecting miR-124-3p in LNCaP and miR-16-5p in both cell lines.

**Conclusion:**

Our findings suggest that this novel approach may have therapeutic benefits and these predicted microRNAs could effectively target the candidate genes.

## Introduction

Prostate cancer (PC) is the most prevalent malignancy in men in 112 countries (1). Despite advances in diagnosis and treatment, PC’s high incidence and mortality rate necessitate novel strategies (2, 3). Many anticancer drugs currently used in clinical practice follow the concept of ‘one molecule - one target - one disease’. However, cancer is a multifactorial disease that may benefit from treatment approaches that target multiple key pathways and/or their pathogenic cross-talk. The multi-target drugs strategy has great potential in cancer therapy as it may simplify treatment regimens, reduce the risk of drug-drug interactions, and most importantly, limit the development of drug resistance.

MicroRNA-based therapies, which are nucleic acid-based therapeutics, have recently been developed and show great potential as a pharmacological platform (4). These therapies utilize small endogenous noncoding RNAs known as microRNAs (miRNAs), which act as post-transcriptional silencing factors. The miRNA’s 5′ seed region binds to the 3′ untranslated region (UTR) of the target mRNA, leading to truncation, destabilization, and turning off of the target mRNA, thereby stopping its translation. 

 Recent research indicates that miRNAs can bind to gene promoters to either activate gene expression or alter protein function, which can be utilized for diagnostic, therapeutic, and prognostic purposes (5-7). Unlike siRNAs, miRNAs only partially bind to target mRNAs through their seed sequence. MiRNAs are important epigenetic regulators in several cancers, including PC, as they can inhibit multiple mRNA targets through a multi-pronged mechanism (8). With the recent progress in miRNA studies, a new generation of anti-cancer drugs that can inhibit multiple pathways is emerging as a significant player.

**Figure 1:**
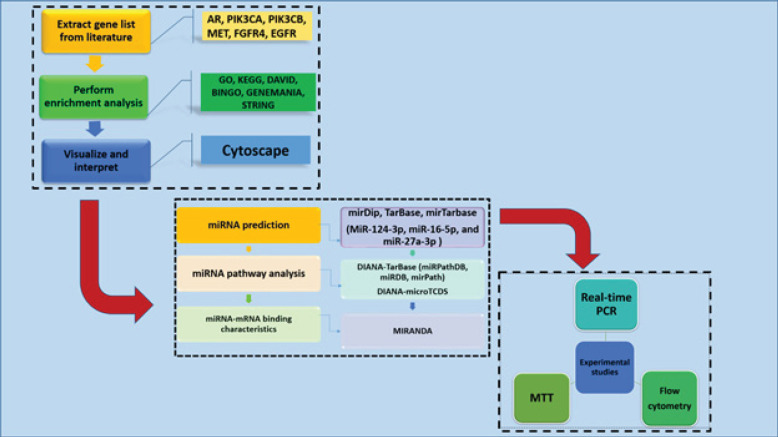
A schematic representation of the workflow of the study in three dashed-line boxes

Pharmaceutical industries have teamed up with bioinformatics to search for multi-target drug design. Computational studies have become increasingly favored as they are more cost-effective and optimize study time. Bioinformatics provides powerful tools for predicting ligand-receptor interactions at the atomic level without requiring extensive experimental setup. Traditional methods of discovering new drug molecules have become outdated due to the prolonged period required for validation through toxicology and pharmacokinetic studies. To develop PC therapy platforms, it is crucial to identify and predict miRNAs that may be relevant. This prediction can be made using web-based bioinformatics tools. There are two main types of miRNA prediction tools: computational algorithms and experimentally validated tools. However, there is no conclusive evidence that one type always outperforms the other. Combining database content has the potential to improve accuracy (9, 10). Developing an effective treatment for PC requires identifying relevant genes that, when blocked, can suppress it. To this end, we selected genes targeted by drugs with clinically proven efficacy and confirmed their protein-protein interaction network using STRING analyses. We also used GO and KEGG pathway analyses to support the role of certain genes in PC. Additionally, we used integrated bioinformatics approaches to identify miRNAs that target the gene set simultaneously and verified the role of specific miRNAs in PC using DIANA-miRPath. Real-time PCR studies showed that these miRNAs suppressed gene expression, and we used flow cytometry to determine where these miRNAs caused the cell cycle to stop. Figure 1 illustrates the study process.

## Materials and Methods

***In silico*** **studies**

*Important drug targets in the development of PC*

Identifying potentially therapeutic miRNAs in PC involves a crucial first step which is selecting suitable drug targets. It’s essential to validate the clinical significance of the chosen genes. For our current investigation, we selected drug-target genes such as *AR, PIK3CA, PIK3CB, MET, FGFR4*, and *EGFR*. These genes have demonstrated clinical efficacy in many clinical trials (11-18). 

*Protein-protein interaction network*

The STRING (https://string-db.org/) web tool was used to acquire insight into the connections between the candidate genes and identify protein-protein interactions (PPI). Both indirect (functional) and direct (physical) connections among proteins were assessed using the medium confidence scores.

*Gene-gene interaction analysis*

GeneMANIA was used to generate a co-expression network by analyzing gene collections and their related genes for interaction (19).

*Enrichment analysis*

In order to determine the clinical significance of the selected genes, we conducted two types of analyses. Firstly, we performed Gene Ontology (GO) enrichment analysis using the Biological Network Gene Ontology tool (BiNGO). Secondly, we conducted a Kyoto Encyclopedia of Genes and Genomes (KEGG) pathway enrichment analysis using the DAVID online tool; this is a web server that can perform functional enrichment analysis on a set of genes (20-23). 

*MiRNA identification using experimentally validated techniques*

Using TarBase v. 837 and miRTarBase v. 816, we predicted gene-miRNA interactions with more accuracy and less false predictions (24). We conducted separate searches in multiple databases for each gene and then tallied up the results of each search. Subsequently, we assembled a list of miRNAs that had multiple gene targets. We ranked these miRNAs based on their validated scores. The validated score for each predicted miRNA was calculated by summing up the number of genes whose targets were identified through empirically validated databases.

*miRNA identification using computational techniques*

Despite their proven reliability, there are only a few experimentally validated tools available for predicting gene-miRNA interactions. Computational tools are more accessible and widely used in comparison to empirical techniques. The most common method to predict gene-miRNA interactions is by identifying seed sequences for complementary nucleotides between miRNA and target mRNA. Online computational methods, such as examining the predicted gene-miRNA duplex’s thermodynamic stability, are frequently used for target prediction algorithms. In order to minimize erroneous predictions and strengthen the accuracy of the predictions made by these algorithms, we utilized more than 30 computational web-based tools, including PITA(25), TargetScan (26), DIANA TOOLS (www.microrna.gr), miRDB (27), and MirTar (28). Each gene was individually searched, and the findings were then combined. A list of miRNAs that could potentially target multiple genes was generated and sorted based on their predicted scores. The predicted score for each miRNA was determined using several computational databases that verified the association between each gene and miRNA. Additionally, MIRANDA was used to examine the characteristics of miRNA-target gene binding.

*Gene and miRNA pathway analysis*

The pathways associated with the target genes were identified using the Kyoto Encyclopedia of Genes and Genomes (KEGG) database, which covers a wide range of genetic, cellular, metabolic, and environmental processes, as well as human disorders.

To study miRNA pathway analysis, we utilized the web tool DIANA-miRPath version 3.0, which can be accessed via http://www.microrna.gr/miRPathv3. This web server integrates several meta-analysis-based miRNA-target interactions and identifies KEGG pathways that are connected to miRNA-target networks. The program uses the DIANA-microT-CDS method, which takes into account the conservation of miRNA-binding sites, to make accurate predictions about miRNA targets.

***In vitro studies***

*Cell culture*

To investigate the impact of miRNAs on target gene expression, we used PC-3 and LNCaP cells. The cells were cultured in RPMI-1640 medium supplemented with 10% FBS under 5% CO_2_ at 37 °C. The cells were procured from the Cell Bank of Pasteur Institute in Tehran, Iran (29). 

*Construction and extraction of plasmid*

The expression vectors pLenti-III-miR16-GFP, pLenti-III-miR124-GFP, and pCDH-miR-27a-GFP, as well as the control vectors pCDH-GFP and pLenti-III-GFP, were purchased from the Stem Cell Technology Research Center located in Tehran, Iran. The next step involved the cultivation of the vector-bearing strain, *Escherichia coli* Stbl4, in the LB medium. Additionally, plasmid DNA was extracted from the overnight-grown *E. coli* using the Qiagen Endo-Free Plasmid Maxi Kit (Qiagen, Hilden, Germany) as per the manufacturer’s instructions.

*Transfection*

LNCaP and PC-3 cells were seeded into 24-well plates with 60000 cells per well and incubated at 5% CO_2_ and 37°C. To achieve transfection, pLenti-III-miR16-GFP, pCDH-miR-27a-GFP, and pLenti-III-miR124-GFP expression vectors along with their corresponding control vectors were transfected into LNCaP and PC-3 cells using PolyFect (Qiagen, Hilden, Germany) as per the manufacturer’s instructions. After 48 hr, fluorescence microscopy was employed to assess the transfection efficiency. Upon successful transfection, the cells were deemed suitable for RNA extraction and qRT-PCR analysis.

*RNA extraction and qRT-PCR*

To extract mRNA and miRNA, cell pellets were first harvested using 0.25% EDTA-trypsin, and an RNX-Plus RNA extraction kit was employed (Sinaclon, Iran). The RNA’s quality and purity were assessed using a BioPhotometer (Eppendorf, Germany). Reverse transcriptase was used to generate cDNA from the whole RNA. Stem-loop primers and oligo dT primers were employed to reverse-transcribe miRNAs and genes, respectively (Table 1). The Roche LightCycler® System was used for analysis, and SYBR Premix Dimer EraserTM (TaKaRa) was used for PCR. Reverse and forward primers for RT-PCR were designed using the AlleleID, OLIGO (Version 7), and Gene Runner primer analysis tools. Each reaction was carried out three times, and the relative levels of miRNA and mRNA expression were normalized and determined using the 2^−ΔΔCT^ method in relation to the SNORD-47 (U47) and GAPDH control genes, respectively (30).

***Cell cycle assay***

PC-3 and LNCaP cells were cultured in 24-well plates with 65000 cells in each well. The cells were treated with 5% CO2 at 37 °C for 24 hr. Next, each cell group was transfected with empty vectors, miR-27a-3p, miR-124-3p, and miR-16-5p in fresh serum-free media. The plates were incubated for 48 hr and then the harvested cells were treated with PI solution. Using flow cytometry by Becton-Dickinson (San Jose, CA, USA), the distribution of the cell cycle was investigated.

*Statistical analysis*

The results of at least three experiments are presented as the mean ± standard deviation (SD). To determine the significance of group differences, one-way ANOVA and Tukey’s *post hoc* analysis were performed using IBM SPSS Statistics V22.0. The data from each experiment were standardized by comparing it to the empty vector treatment, and the significance of the results was then evaluated. Furthermore, using the Kolmogorov-Smirnov technique, normality test analysis was conducted in SPSS. The significance values for differences were denoted by *P<0.05.

## Results

***Drug-target genes***

We selected genes that target drugs that have been clinically shown to treat PC, to identify those which may inhibit PC. In this research work, candidate genes included *AR, PIK3CA, PIK3CB, MET, FGFR4,* and *EGFR* (Table 2). 

***PPI analysis***

**Figure 2:**
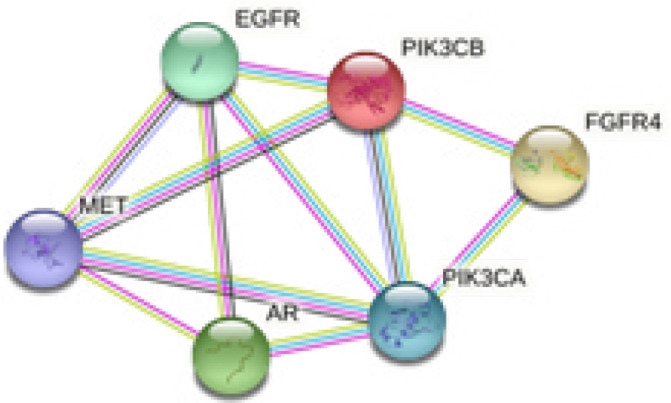
The network of protein-protein interactions (PPIs) depicts the interactions between the selected genes' proteins

The STRING web tool was used to demonstrate the network of interactions between candidate genes. The network presents each gene as a node, and it uses distinct colors to represent different clusters and edges to represent the functional relationships that exist between genes. Additional evidence is used to suggest which edges are likely to exist, and the predicted connections are further distinguished. Figure 2 shows the PPI network with a medium degree of confidence.

***Gene-gene interaction analysis***

**Figure 3:**
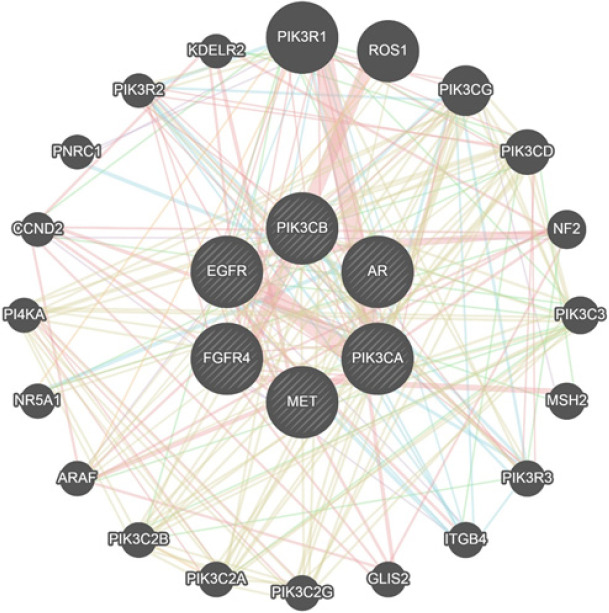
The co-expression network of the selected genes was generated using the GeneMANIA plugin in Cytoscape

After selecting specific genes, we used GeneMANIA’s vast functional interaction datasets to create a co-expression network. Figure 3 illustrates 20 nodes surrounding our target genes. We also identified several other genes that were connected to the chosen set of genes. Among these genes, the top five crucial genes that showed the strongest correlation with our targets were ROS proto-oncogene 1, phosphoinositide-3-kinase regulatory subunit 1 (PIK3R1), phosphatidylinositol-4,5-bisphosphate 3-kinase catalytic subunit delta (PIK3CD), receptor tyrosine kinase (ROS1), phosphatidylinositol-4,5-bisphosphate 3-kinase catalytic subunit gamma (PIK3CG), and neurofibromin 2 (NF2). 

***Enrichment analysis***

**Figure 4:**
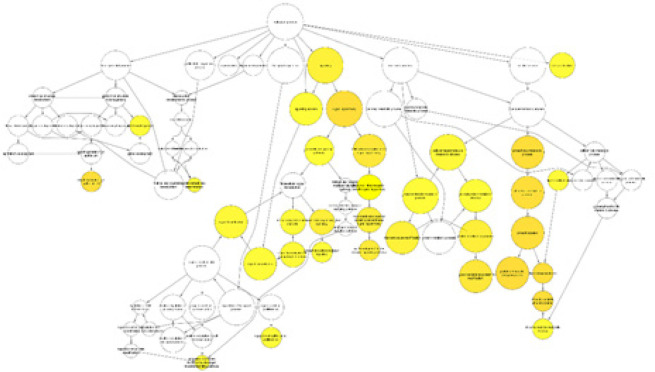
TThe BINGO analysis of six potential genes reveals a biological mechanism

The Cytoscape BiNGO Plugin was utilized to identify crucial genes involved in the pathophysiology of PC. This tool employs Java to identify genes with significantly overrepresented Gene Ontology (GO) categories. Each node in the analysis represents a biological process, with darker shaded nodes indicating the most noteworthy processes. These processes include signaling pathways, cell growth, EGF, and AR pathways, post-translational protein modification, phosphorylation, and more (Figure 4).

DAVID analysis revealed that six genes in PC were overrepresented. This platform utilizes DISGENET, KEGG, Reactome, and GO for forecasting signaling pathways and complex biological processes. As per FDR, Table 3 lists the top three enriched phrases. All six candidate genes are included in the top-ranked enriched terms. The FDR was set to 0.05 to detect enriched terms.

**Figure 5:**
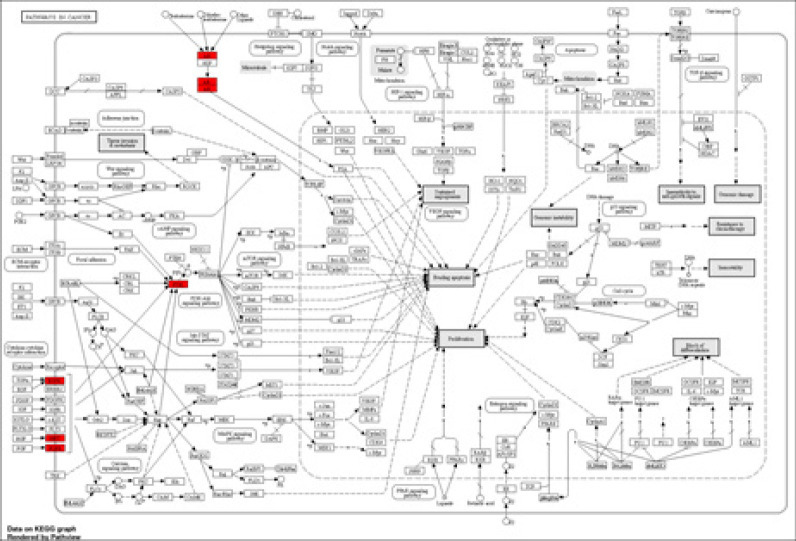
The KEGG analysis results

KEGG pathway enrichment analysis yielded a network of six candidate genes for PC (Figure 5). 

***MiRNA identification using experimentally validated and computational tools***

We compiled a list of the three most promising miRNAs that were predicted and experimentally verified to target specific genes. Using advanced algorithms, we were able to identify three miRNAs for each of the six targeted genes. Among the top-scoring miRNAs were hsa-miR-27a-3p, 124-3p, and 16-5p, which were then selected for further analysis (Table 4).

Additionally, MIRANDA was used to determine the location, region, and free energy of miRNA-mRNA interactions. Table 5 shows the binding pattern and the resultant parameters. Only the top three miRNA-mRNA interactions are displayed, ranked by free energy.

***Pathway analysis***

**Figure 6:**
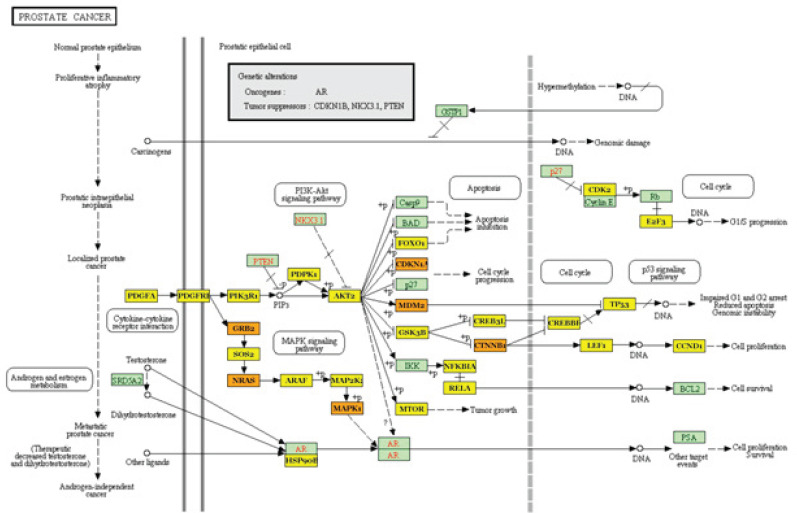
miR-27a-3p, miR-124-3p, and miR-16-5p target genes participate in PC-related pathways

We used DIANA-miRPath to identify miRNA-gene interactions related to PC pathways. The analysis revealed that miR-16-5p, miR-27a-3p, and miR-124-3p are associated with pathways related to PC. These miRNAs target genes that play a crucial role in androgen metabolism, which is a key process in PC (Figure 6).

***Overexpression of miR-27a-3p, miR-16-5p, and miR-124-3p***

**Figure 7:**
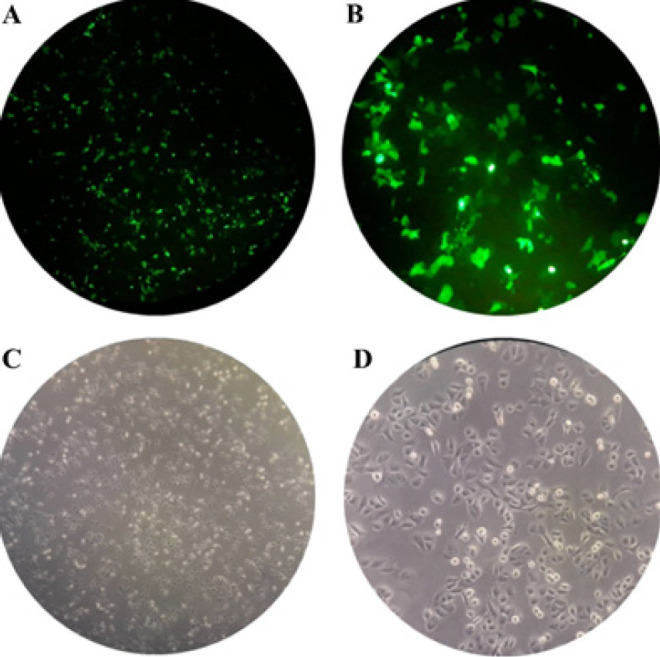
GFP expression of plasmids used as a reporter for miRNA expression in PC3 and LNCap cells

**Figure 8:**
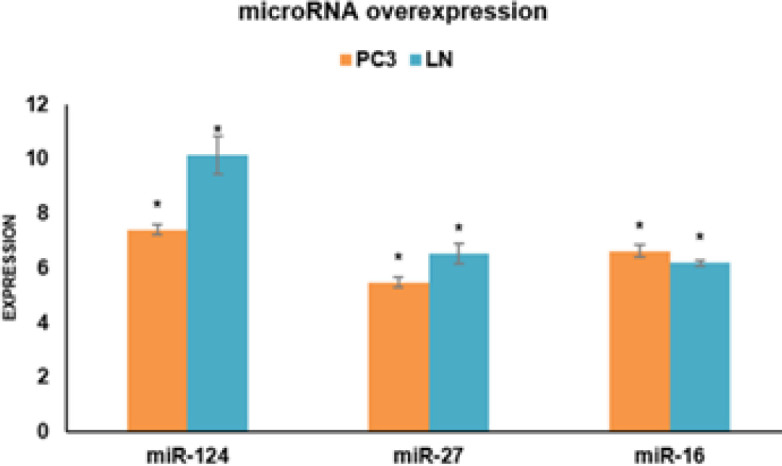
Following transfection with miR-27a-3p, miR-16-5p, and miR-124-3p, gene expression was measured for 48 hr

Transfection efficiency was determined by the GFP-expressing plasmid. The presence of GFP-expressing cells was detected through fluorescent microscopy. To confirm the effects of miR-27a-3p, miR-16-5p, and miR-124-3p in LNCaP and PC-3 cells, the empty vectors or plasmids encoding these miRNAs were transfected. Optimal expression was observed 48 hr later through GFP signal monitoring (Figure 7). Subsequently, overexpression of miR-27a-3p, miR-16-5p, and miR-124-3p was confirmed through qRT-PCR. We found that the candidate miRNAs were significantly overexpressed in both PC-3 and LNCaP cells (*P<0.05) (Figure 8).

***Effect of the selected miRNAs on target gene expression in LNCaP cells***

**Figure 9:**
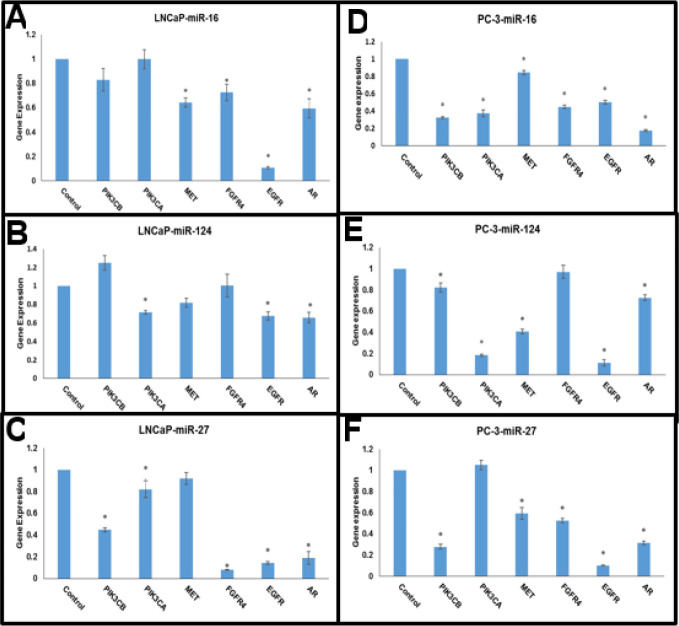
Here are the results of gene expression 48 hr after transfection

Following the transfection of either empty control vectors or miR-16-5p, miR-124-3p, and miR-27a-3p in the LNCaP cells, the expression of target genes was measured using qRT-PCR. The results showed that miR-16-5p significantly down-regulated *AR, EGFR, FGFR4*, and *MET*, miR-124-3p down-regulated *AR, EGFR*, and *PIK3CA*, while miR-27a-3p down-regulated *AR, EGFR, FGFR4, PIK3CA*, and *PIK3CB* relative to the control group (*P*-value<0.05) (Figure 9 A, B, and C).

***Effect of the selected miRNAs on target gene expression in PC-3 cells***

After transfecting the PC-3 cell line with miR-16-5p, miR-27a-3p, miR-124-3p, and empty control vectors, we measured the expression of target genes using qRT-PCR. Our findings revealed that miR-16-5p significantly down-regulated the entire gene set, while miR-124-3p down-regulated all genes except for *FGFR4*. On the other hand, miR-27a-3p significantly down-regulated the entire gene set except for *PIK3CA*, as compared to controls (*P*-value<0.05) (Figure 9 D, E, and F).

***Effect of miR-124-3p, miR-27a-3p, and miR-16-5p on cell cycle distribution in PC-3 cells***

**Figure 10:**
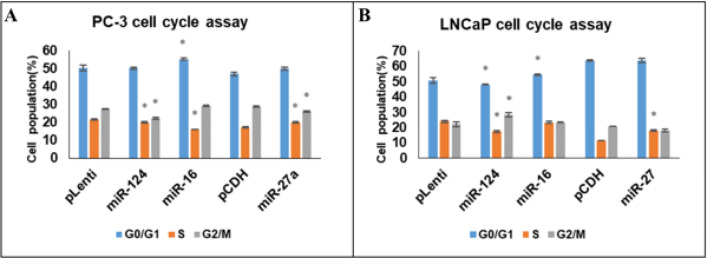
Cell cycle analysis was performed 48 hr post-transfection with miR-124-3p, miR-27a-3p, and miR-16-5p

The distribution of cell cycle in PC-3 cells transfected with miR-16-5p, miR-124-3p, 27a-3p, and empty vectors was investigated using flow cytometry. All miRNA treatments stopped the S-phase, but only miR-16-5p arrested the G0/G1 phase. The G2/M arrest was significant in all cases except for miR-16-5p. The results are presented as means ± SD with a *P*-value of less than 0.05 (Figure 10, A).

***Effect of miR-124-3p, miR-27a-3p, and miR-16-5p on cell cycle distribution in LNCaP cells***

Using flow cytometry, we investigated the distribution of the cell cycle in LNCaP cells that had been transfected with either miR-16-5p, miR-124-3p, and 27a-3p or empty vectors. We found that miR-124-3p and miR-16-5p caused G0/G1 arrest and miR-124-3p and miR-27a-3p treatment led to S-phase arrest. However, only miR-124-3p treatment resulted in a significant G2/M arrest. Our findings are presented as mean ± standard deviation (*P*<0.05) (Figure 10, B).

**Table 1:** For qRT-PCR and cDNA synthesis, F and R primers were used for each miRNA or gene, along with oligo-dT and specific stem-loop primers, respectively. R (reverse primer), F (forward primer)

Name	Primer sequence (5′-3′)
hsa-miR-124-3p	RT primer: GTCGTATGCAGAGCAGGGTCCGAGGTATTCGCACTGCATACGACTTGGCA
	F primer: CTAAGGCACGCGGTGAA
hsa-miR-16-5p	RT primer: GTCGTATGCAGAGCAGGGTCCGAGGTATTCGCACTGCATACGACCGCCAA
	F primer: GCCTAGCAGCACGTAAATA
hsa-miR-27a-3p	RT primer: GTCGTATGCAGAGCAGGGTCCGAGGTATTCGCACTGACGACGCGGAA
	F primer : CCGTTCACAGTGGCTAAG
SNORD 47	RT primer: GTCGTATGCAGAGCAGGGTCCGAGGTATTCGCACTGCATACGACAACCTC
	F primer: ATCACTGTAAAACCGTTCCA
Universal Revers miR	GAGCAGGGTCCGAGG
AR	F primer: GACATGCGTTTGGAGACTG
	R primer: CAATCATTTCTGCTGGCGC
PIK3CA	F primer: CTCCTCTAAACCCTGCT
	R primer: CATATCTTGCCGTAAATCATCCC
PIK3CB	F primer: TGCGACAGATGAGTGATGAAGAA
	R primer: AACTGCCCTATCCTCCGATTAC
MET	F primer: GCTAATCTTGGGACATCAG
	R primer: ATCTTCGTGATCTTCTTCC
FGFR4	F primer: CTTGATTACAGGTGACTCC
	R primer: TGGACAGCGGAACTTGAC
EGFR	F primer: CGCAAAGTGTGTAACGGAATAGG
	R primer: AGAGGAGGAGTATGTGTGAAGG
GAPDH	F primer: CCTCAAGATCATCAGCAATG
	R primer: CATCACGCCACAGTTTCC

**Table 2:** Based on clinical studies, there is supporting evidence for the importance of the selected crucial genes

Symbol	Gene name	Drug	Pathway	Alterations
*AR*	Androgen receptor	Xtandi/MDV3100/enzalutamide, ODM-201, ARN509	Androgen receptor signaling	Amplification, Mutations, Variant splicing
*PIK3CA & PIK3CB*	Phosphatidylinositol-4,5-bisphosphate 3-kinase catalytic subunit alpha & beta	BKM120, GDC0980, GSK2636771, BEZ235	PI3K signal transduction, Co-operates with the AR pathway in the pathogenesis of PCa	Overexpression, Mutations
*MET*	*MET* proto-oncogene, receptor tyrosine kinase	Cabozantinib /XL184, Tivantinib ARQ 197, Onartuzumab	Growth factor-induced signaling, activation of PI3K and MAPK pathways, and AR signaling	Activation
*FGFR4*	Fibroblast growth factor receptor 4	Dovitinib/TKI258	Developmental pathways, Growth factor-induced signaling, activation of PI3K and MAPK pathways, and AR signaling	Overexpression, Activation
*EGFR*	Epidermal growth factor receptor	BIBW 2992/Afatinib, Lapatinib, PLX3397	Growth factor-induced signaling, activation of PI3K and MAPK pathways, and AR signaling	Activation

The gene targets have been selected, and their interaction network was confirmed using STRING, GO, and KEGG (upper box); using integrated bioinformatics approaches, candidate miRNAs were identified (middle box). Real-time PCR and flow cytometry showed that these miRNAs suppressed gene expression and caused the cell cycle to stop (lower box).

The lines representing evidence of co-occurrence, text mining, fusion, experimental validation, co-expression, database records, and neighborhood are colored blue, yellow, red, purple, black, light blue, and green, respectively. The thickness of a line indicates the strength of the evidence. The edges illustrate the connections between proteins and their predicted functional relationships, while the circles represent the proteins expressed by the relevant genes

The target proteins were represented by the black nodes, while the different colors of the links indicated diverse associations. The genes inside the central circles that are crosshatched refer to the query terms, while the black genes represent those associated with the query genes. Purple connections indicate co-expression, while gold lines represent predictions. Blue lines depict various pathways, and green lines indicate conserved protein domains.

The node size corresponds to the number of genes implicated in the process, while the darker color signifies a smaller *P*-value. Statistical significance is demonstrated by colored nodes, which usually have a *P*-value lower than 0.05. White nodes function as intermediaries between biological processes but do not possess statistical significance.

After conducting a KEGG pathway enrichment analysis, a network of six potential genes for PC was identified. For improved visibility, the candidate genes have been highlighted in red.

**Table 3:** DAVID analyzed the GO and KEGG pathways. Enriched terms were organized by FDR. Terms with FDR < 0.05 were considered significant

Category	Term	Count	FDR
**DISGENET**	Malignant neoplasm of the prostate	6	1.12E-04
**DISGENET**	Prostatic neoplasms	6	1.12E-04
**KEGG_PATHWAY**	Pathways in cancer	6	3.04E-05

**Table 4:** After analyzing the data from mirDip, TarBase v8, and miRTarbase, we were able to identify potential miRNAs

miRNA	Integrated score	Number of sources	Number of predicted genes	Positive evidence	Negative evidence	Net	Number of validated genes
Hsa-miR-124-3p	1.568098	32	6	7	2	5	6
Hsa-miR-16-5p	1.0621409	26	6	15	1	14	6
Hsa-miR-27a-3p	1.6773427	39	4	6	2	4	5

**Table 5:** Characteristics of miRNA-mRNA interactions that create a strong connection

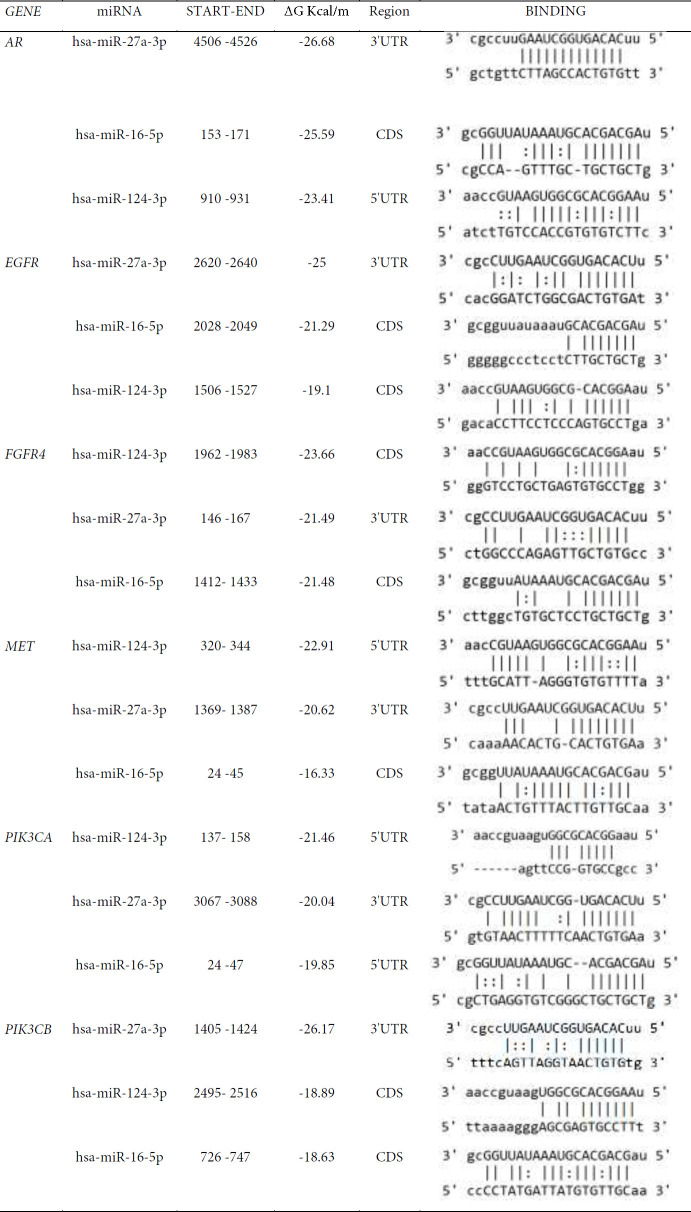

A & B, fluorescence microscopy imaging; C & D, light microscopy imaging at 48 hr post-transfection

Overexpression of miR-27a-3p, miR-16-5p, and miR-124-3p was observed in LNCaP and PC-3 cells at 48 hr post-transfection (*P<0.05).

The impact of miR-16-5p, miR-124-3p, and miR-27a-3p on target gene expression was observed in LNCaP cells (A, B, and C) and PC-3 cells (D, E, and F), respectively. The data presented shows the mean ± SD of at least three replicates. We used one-way ANOVA and Tukey's post-hoc analysis to evaluate statistically significant differences between the groups. The control vector was used to determine statistical significance (*P<0.05).

The proportion of the cell population was compared to controls (mean ± SD from three separate trials; *P<0.05).

## Discussion

Drug discovery involves the development of ligands that selectively target specific drug targets. However, for complex diseases such as cancer, a single medication targeted at a single activity may not be sufficient. MiRNA-based therapeutics can modulate the expression of numerous disease-related genes, making them a powerful tool for fighting diseases. However, miRNA interactions with mRNA are not yet fully understood and miRNAs may have off-target effects on other pathways. In this work, we used a combined bioinformatics approach to address this issue. Over the past few years, various miRNA-gene interaction prediction databases and algorithms have been established. Although experimentally verified databases may be more accurate, there is not enough empirical data to develop them. Computational approaches can help determine the association between compounds and targets before any chemical synthesis or biological testing but depend on prior identification of clinically and biologically validated targets. Combining validated and predicted techniques can limit off-target effects and help identify the best miRNAs for therapy.

Multi-target drugs are being developed to treat complex diseases like cancer that require therapies that address multiple targets. This shift in cancer therapy is due to the specificity of the disease. To predict anti-PC miRNAs against several PC cell lines, a new approach using a multi-target strategy has been presented. The study uses six drug target genes found in clinical trials—*AR, PIK3CA, PIK3CB, MET, FGFR4*, and *EGFR*—to identify effective miRNAs. These genes’ strong protein-protein interactions were validated through STRING analysis. The miRNAs miR-124-3p, miR-27a-3p, and miR-16-5p were found to have the potential to target the candidate gene set simultaneously. Bioinformatics findings were confirmed through *in vitro* evaluation of miR-124-3p, miR-27a-3p, and miR-16-5p.

A recent study by Kalofonou *et al.* found that miR-27a-3p acts as a modulator for the *AR* (31). Further research has shown that miR-27a-3p plays a crucial role in regulating the epithelial-mesenchymal transition, tumor immune system response, and chemoresistance (32). Targeting *EGFR*, which is a direct target of miR-27a-3p, could potentially help reduce PC (33). Additionally, it has been found that miR-27a-3p inhibits invasion and proliferation both *in vivo *and* in vitro* (34). In this regard, our results indicate that miR-27a-3p down-regulates almost all examined genes in both cells.

Our study revealed that the expression of miR-124-3p led to the significant suppression of *PIK3CA, FGFR4*, and *AR* genes in LNCaP cells, while in PC-3 cells, it suppressed all genes except *FGFR4*. MiR-124-3p has been identified as a prognostic marker for PC, and its prognostic value is independent of other factors (35). Research has suggested that the development of PC is inhibited by miR-124-3p through the targeting of *AR* and *p53* (36-38). It is reported that PC cells express less miR-124-3p than normal cells, and their onset and development may be associated with the decline in miR-124-3p (39). Shi *et al*. also observed that PC often declines miR-124-3p expression (37). Previous studies have demonstrated that miR-124-3p overexpression altered the cell population’s percentage in human PC cell lines. Wu and colleagues’ study reported that miR-124-3p overexpression impedes the proliferation and migration of PC cells and induces G0/G1 cell cycle arrest, thus inhibiting apoptosis (40). Our study suggests that miR-124-3p may be capable of slowing down the progression of PC. Hence, miR-124-3p overexpression could be considered a potential treatment option for PC.

Our research has revealed that miR-16-5p targets almost all of the selected genes. Consistent with previous studies, miR-16-5p arrested the cell cycle in the G0/G1 phase (41). These findings imply that miR-16-5p has a suppressive effect on tumors (42). In addition, the majority of Musumeci *et al.*’s 23 PC patients showed down-regulation of miR-16-5p. Furthermore, it has been reported that various types of cancer display down-regulation of this miRNA (43). A study discovered that miR16 blocked TGF-β signaling pathways in LNCaP cells, which may have an impact on the growth and metastasis of PCs (44). Overall, previous research suggests that miR-16-5p acts as a tumor suppressor in PC, controlling cell invasion, proliferation, and survival, which is consistent with our findings (45). 

Since we had experimental limitations, we need to uncover how these miRNAs inhibit protein levels in the target genes. In addition, more *in vivo* and animal studies are necessary to discover the therapeutic and adverse effects of these three miRNAs. In addition, *in vivo*, delivering miRNAs to target sites could minimize toxicity and side effects, as well as enhance the efficacy of treatment.

## Conclusion

Our study aimed to address the limitations of current PC treatments. We identified three microRNAs, namely miR-124-3p, miR-27a-3p, and miR-16-5p, which can effectively target up to six key genes in PC. These candidate genes are crucial for PC, and reducing their expression could potentially treat the disease. The inhibitory effects of these microRNAs were also confirmed *in vitro*. However, further research is required to validate these findings. 

## Authors’ Contributions

SH AB, A J, and R KO contributed to the study conception and/or design. S A and MH JN performed the experiment and analysis and interpretation of results. S A and MJ B helped with draft manuscript preparation and visualization. SH AB, A J, and R KO conducted the critical revision of the article. S A, MH JN, MJ B, A J, R KO & SH AB approved the final version to be published. SH AB supervised and acquired funds.

## Conflicts of Interest

No declarations were made.
